# Mortality in Newly Admitted Nursing Home Older Adults with Dementia in France: A Post Hoc Analysis from an Observational Study in the Bordeaux Region

**DOI:** 10.3390/geriatrics9060149

**Published:** 2024-11-13

**Authors:** Damien Krier, Mélanie Le Goff, Catherine Helmer, Jérôme Wittwer

**Affiliations:** Bordeaux Population Health Research Center, University of Bordeaux, INSERM U1219, 146 Rue Léo Saignat, F-33076 Bordeaux, France; melanie.le-goff@u-bordeaux.fr (M.L.G.); catherine.helmer@u-bordeaux.fr (C.H.); jerome.wittwer@u-bordeaux.fr (J.W.)

**Keywords:** long-term care, mortality, evaluation, dementia, Alzheimer’s disease

## Abstract

Background/Objectives: A significant proportion of older adults with Alzheimer’s disease or related disorders live in a long-term care facility. This study aimed to determine the time delay between admission and death for older adults with dementia. Methods: A post hoc analysis was conducted using data from a French observational cohort, identifying older adults with dementia who were admitted to nursing homes. This study assessed median survival times after admission to care facilities by using Kaplan–Meier models and evaluated factors potentially associated with the time until death by using Cox models. Results: A total of 201 individuals were included. The median survival time from admission to a nursing home to death was 39 months. Being male, an older age, and having higher cognitive impairment and comorbidities were associated with decreased survival rates. Conclusions: This study provides survival results for institutionalised older adults with dementia in France and provides elements for the definition of future public policies.

## 1. Introduction

As the global population continues to age, the prevalence of Alzheimer’s disease and related disorders (ADRD) has garnered increasing attention [1]. In Europe alone, approximately 10 million people are currently living with dementia, and this number is expected to double by 2050 [2]. Dementia impairs both cognitive and functional abilities and is often associated with physical health conditions such as cardiovascular disease and infections, which significantly contribute to increased mortality rates [3]. Moreover, recent studies have emphasised the impact of frailty on dementia progression and mortality [4]. In major Western countries, it is estimated that between 33% and 50% of individuals with dementia live in nursing homes (NHs) [5]. It is well established that advanced dementia is the primary cause of institutionalisation [6,7]. For most individuals, the transition from home to institutional care is a critical period, often associated with negative outcomes, including increased mortality [8,9,10]. Despite recent advances in understanding AD, treatment options remain limited, and optimising care and support for older adults and their families continues to be a major public health challenge [11,12].

NHs play an important role in end-of-life care and are often the place where people spend their last days [13]. The proportion of deaths occurring in these facilities has increased in recent years [14]. In France, nearly one in ten people aged 75 and older reside in a residential care facility, and this proportion doubles to 21% after the age of 85 [15]. Most of these individuals live in NHs known as ‘Établissement d’hébergement pour personnes âgées dépendantes’ (EHPADs), a term used for residential facilities that provide care for elderly people who are dependent on assistance with daily activities and medical care. NHs offer a wide range of services, including accommodation, meals, nursing care, and leisure activities.

The time spent in an NH before death provides valuable clinical insights and guidance for public policy, particularly in planning interventions to prevent early admission to NHs. A recent study in Norway estimated the median survival of individuals with dementia admitted to NHs to be 2.3 years [16]. A German study compared the median survival time between admission to an NH and death for residents with and without dementia; it was 2 years overall, 25 months for those with dementia, and 21 months for those without [17]. In France, an analysis by DREES (Direction de la Recherche, des Études, de l’Évaluation et des Statistiques) estimated the median survival time from admission to death for newly admitted residents (not specific to older adults with dementia) as 31 months [13]. Few studies have assessed the mortality of residents with dementia newly admitted to an institution, particularly in France. There are very few data available in France, especially on people with dementia, which are essential for planning care. Additionally, there are no available findings on the determinants of mortality, which are essential for designing and implementing effective interventions.

The aim of this study was to estimate the survival time of residents with dementia newly admitted to an NH and to analyse the factors associated with mortality.

## 2. Methods

### 2.1. Study Sample

This study was conducted within the Three-City (3C) population-based cohort, which included individuals aged 65 years and older. The primary objective of the 3C study, a multicentre study, was to estimate the risk of dementia attributable to vascular risk factors. Between January 1999 and February 2001, a total of 9294 noninstitutionalised individuals aged 65 and older from three French cities—Bordeaux (*n* = 2104), Dijon (*n* = 4931), and Montpellier (*n* = 2259)—were enrolled [18]. The participants in Bordeaux were followed for almost twenty years, until 2018, while the follow-up in the other centres was shorter—up to 12 years in Dijon and 15 years in Montpellier. Vital statuses and dates of death were regularly assessed throughout the follow-up period. Only participants from the Bordeaux centre were included in this paper, as it is the only centre where the place of residence (in a nursing home or not) was collected. Up to seven follow-up visits were conducted at the participants’ homes by a psychologist using a standardised questionnaire that collected sociodemographic data (age, sex, education level, etc.) and clinical data (cognition, functional abilities). The diagnosis of dementia was made according to the Diagnostic and Statistical Manual of Mental Disorders (DSM-IV, 4th edition), following a three-step procedure: initial screening by a psychologist, examination by a neurologist or geriatrician, and a review of all files by a committee of independent experts.

**Figure 1:**
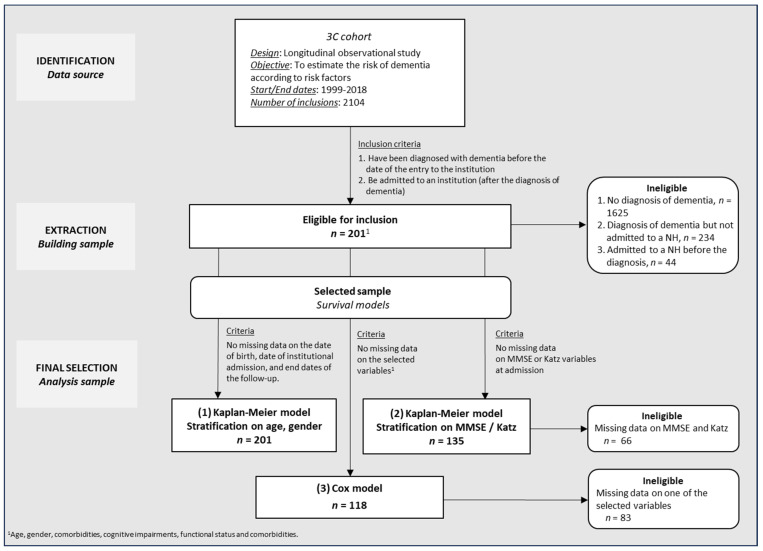
Flowchart, sample selection for analysis.

Participants had to meet the following criteria to be included in this post hoc analysis: (1) be diagnosed with dementia during the first 14 years of the study; (2) be admitted to an NH after being diagnosed with dementia. Of the 2104 participants enrolled at the Bordeaux centre, 479 individuals were diagnosed with dementia or developed it during the follow-up period, and 245 were admitted to an NH. Among them, 44 were admitted to a nursing home before receiving a diagnosis of dementia. The remaining 201 individuals, whose date of admission to an NH was after their diagnosis of dementia, formed the analysis sample. The selection process is presented in Figure 1.

### 2.2. Data Availability

The 3C study included several variables, such as sociodemographic characteristics (age, sex, educational level, and marital status) and clinical data (number of medications, comorbidities/history, cognition, and functional status). Clinical characteristics at enrolment were defined based on the assessment closest to the date of NH admission, conducted within 12 months before or after that date. Specifically, for clinical characteristics assessed using the Katz Index of Independence in Activities of Daily Living (ADL), the Lawton Instrumental Activities of Daily Living (IADL) scale, and the Mini-Mental State Examination (MMSE), data corresponded to assessments performed at the time of admission or within 12 months before or after admission. The Katz ADL scale evaluates a person’s ability to perform six essential daily activities without assistance or supervision (each activity is scored as 0—independent, 1—partially dependent, or 2—dependent). In this study, the Katz score calculation excluded the ‘incontinence’ item, resulting in a score range from 0 (totally independent) to 10 (totally dependent). The decision to use a five-item ADL scale, excluding continence, for elderly people in residential care was based on clinical reasoning. The exclusion of continence is justified by the fact that incontinence is not directly linked to dependency, as an individual can be incontinent yet still independent. Furthermore, incontinence is considered an impairment rather than a disability, meaning that while it may pose a challenge, it does not necessarily result in a loss of autonomy as a person can manage his/her incontinence him/herself [19,20]. In this study, a score of 4 or higher on the Katz scale indicates a significant level of dependence, while a score below 4 reflects a relatively higher level of independence. During the 3C study visits, various types of information regarding the participants’ health and key medical history were collected. The presence of any of the following conditions was assessed: diabetes, cancer, hypertension, cardiac rhythm disorders, peripheral arterial disease, heart failure, and hypercholesterolaemia. If a participant was unable to respond, the study psychologist obtained information from the caregiver or medical staff.

### 2.3. Statistical Analysis

The Kaplan–Meier method was used to construct survival curves and estimate median survival. The primary advantage of this method is that it does not require data from regular time intervals and can analyse the evolution of survival status in the population over time [21]. Median survival was estimated for individuals with dementia admitted to NHs. The observation period for participants was calculated as the time elapsed between the date of NH admission and the end of the follow-up. The end-of-follow-up date was defined as either the date of death or the date of the last follow-up, and the observation period was measured in months. Survival analysis was stratified by sex, age, and clinical characteristics at admission, including cognitive function, functional ability, and comorbidities. A multivariate analysis using Cox proportional hazard models was conducted to identify factors associated with mortality [22].

The selection of variables for inclusion in the Cox model followed a multistage process. Initially, this selection was guided by domain knowledge, focusing on factors likely to influence survival based on the available variables. Preliminary analyses, both univariate and multivariate, were conducted to assess the effect of these variables on mortality. An iterative process was then used to eliminate variables that did not appear to influence mortality, resulting in five key variables of interest: age, sex, cognitive status, comorbid hypertension, and the number of comorbidities at admission.

## 3. Results

### 3.1. Samples Description

A total of 201 participants met the inclusion criteria and were included in the analysis.

Three analysis populations were used to implement the different methods:

- KM modelling on the whole sample, followed by stratification according to age and sex;
- KM modelling on the whole sample, followed by stratification according to cognitive and functional score, MMSE, and Katz;
- Cox modelling on selected variables.

The population extracted from the cohort was used to model resident survival for all individuals, including 201 older adults. Analyses were also performed by stratifying by sex and age at admission. A description of the sample is given in Table 1, and includes the sociodemographic and clinical characteristics available at the time of admission to the facility. For the clinical characteristics (Katz, Lawton, and MMSE), data were based on an assessment close to the time of admission, i.e., in the 12 months preceding or in the 12 months following admission. Of the initial sample of 201, 56 participants were not seen within 12 months before or after their admission. They therefore had missing data for all clinical variables (cognitive and functional status). The majority of the sample was female (80%). The minimum and maximum ages at admission to NHs were 71 and 96 years, respectively, with a mean of 86 (SD = 5), which did not differ between women. Of the comorbidities assessed, 31% of individuals had no comorbidities, while more than half were affected by one or two comorbidities. Hypertension affected almost 50% of the residents (*n* = 93), and a quarter were affected by at least one cardiovascular disease (28%, *n* = 51), mainly including cardiac rhythm disorders (*n* = 38) and heart failure (*n* = 20). Hypercholesterolaemia and diabetes affected 20% and 13%, respectively. Severe cognitive impairment (MMSE score < 10) was present in 16% of residents. The Katz ADL scale assesses a person’s ability to perform six essential activities of daily living without assistance or supervision (each activity is scored as either 0—independent or 2—dependent). The calculation of the Katz score did not include the ‘incontinence’ item from the Katz scale, so the score could range from 0 (total independence) to 10 (total dependence). The decision to use a five-item ADL scale, excluding incontinence, for older people in residential care is based on clinical reasoning. Using a score of 4 as the threshold to define dependence or independence status, approximately 55% of individuals were independent.

Further univariate survival analyses of median survival duration were conducted based on the level of cognitive impairment and functional capacity at admission. By retaining only individuals who had assessments of MMSE and Katz scores within 12 months after admission, this subsample included 135 individuals. This subsample showed similar descriptive statistics to the full sample (see Supplementary Materials).

In the case of this study, only clinical data on cognitive impairment (MMSE score) and functional status (Katz score) were available. Other available variables included in the model were sex, age, comorbidities, or medical history (e.g., cancer). Preliminary Cox analyses showed no effect of these variables on mortality; therefore, they were excluded from this analysis to maximise the sample size. To perform analyses using Cox models, individuals with missing data on any of the selected variables were excluded. In the end, 108 participants remained for the analyses. Descriptive statistics for this sample are not detailed in this paper, but the distributions of the various variables were relatively similar to those described in the previous population (*n* = 135) in terms of sex, age, cognitive impairment, and level of dependency. Univariate survival analyses were also conducted on this subsample, with stratification by age and sex. These survival analyses yielded the same results as those for the initial KM initial sample (1).

### 3.2. Survival Analysis

#### 3.2.1. KM Models with Age and Gender Stratification

**Figure 2:**
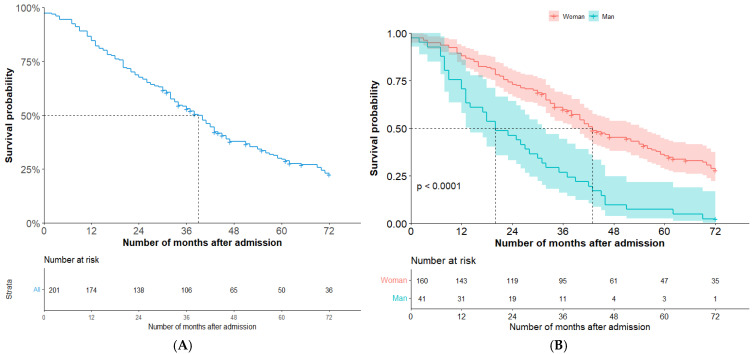
Survival curve for older adults with dementia newly admitted to a nursing home (**A**), and by gender (**B**).

Of the 201 individuals included in the analysis sample, a total of 147 residents died during the study. Follow-up occurred between 2000 and 2018, with the first and last admissions to nursing homes taking place on 16/02/2000 and 09/07/2015, respectively, resulting in a maximum follow-up period of nearly 17 years. For individuals included in the analysis, median survival was 39 months with a 95% confidence interval (CI) of 33 to 43 months, and the survival curve is shown in Figure 2A. As shown in Figure 2B, female residents spent longer in nursing homes, with a significant difference in median survival time compared to males. The median time from admission to death was 43 months for females (95% CI 38–55, *n* = 160) and 20 months for males (95% CI 14–31, *n* = 41). Survival was slightly shorter for those admitted at an older age, i.e., those aged 65 to 86 years (*n* = 118), compared to those over 85 years (*n* = 83), with median survival times of 42 months and 36 months, respectively, though the difference was not significant (*p* = 0.06).

#### 3.2.2. KM Models with Stratification by Functional and Cognitive Status

A survival analysis was conducted based on cognitive impairment and functional status at enrolment, including individuals who had both MMSE and Katz score assessments (*n* = 135). For those included in the analysis, the median survival was 40 months (95% CI 36–45) (see survival curve in Supplementary Material Figure S1). Individuals with severe cognitive impairment (MMSE score ≤ 10, *n* = 21) appeared to have a shorter median survival compared to those with mild to moderate cognitive impairment (*n* = 114), with 32 months and 41 months, respectively, although the difference was not statistically significant (*p* = 0.33). Survival time was calculated according to dependency at admission. Being independent at admission is potentially not associated with better survival in an NH, with a median of 40 months for independent individuals (95% CI 36–47, *n* = 73) and for dependent individuals (95% CI 32–46, *n* = 62).

#### 3.2.3. Cox Model

**Figure 3:**
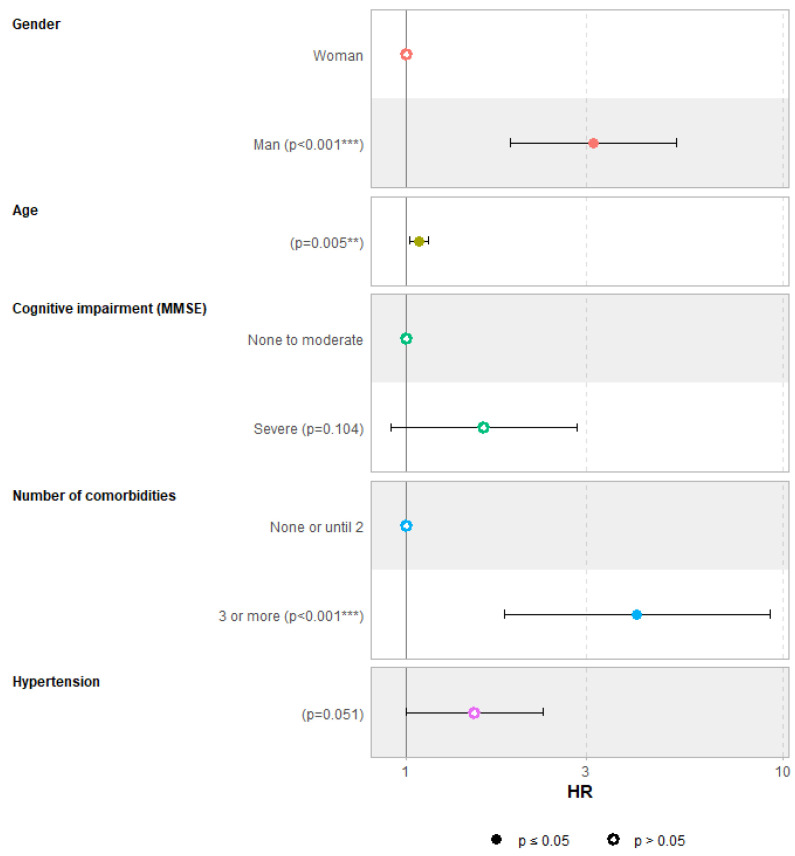
Factors associated with mortality among older adults with dementia newly admitted to a nursing home, graphical representation of the results of the Cox model (*n* = 118). *p* < 0.01 (**), *p* < 0.001 (***).

The analysis was conducted on 118 individuals, excluding 83 participants who had missing data for at least one of the selected variables. The model estimates the coefficients of the explanatory variables and their effect on the risk of death. The results are expressed as instantaneous hazard ratios (HRs) with 95% confidence intervals and are presented in Figure 3 (see Supplementary Materials for the results table).

Coefficient estimation highlights several explanatory variables that positively influence the risk of death. A resident’s age at admission is a significant factor, with an increase in age being associated with a higher risk of death (HR = 1.08; *p* < 0.01), meaning the risk increases by 1.08 for each additional year. Sex also appears to influence mortality, with males having a significantly higher risk of death (HR = 3.13; *p* < 0.001). In the Kaplan–Meier (KM) model, older individuals with severe cognitive impairment (MMSE < 10) were at greater risk of death compared to those with mild to moderate impairment. After adjustment for demographic characteristics and other clinically relevant factors (comorbidities and functional status), a similar association between cognitive impairment and mortality was observed. A high level of cognitive impairment at the time of admission increased the risk of death by 1.61, even if the result is on the verge of significance (*p* = 0.10). Lastly, having three or more comorbidities significantly increased the risk of death, with an almost threefold higher risk compared to participants with two or fewer comorbidities.

## 4. Discussion

This study is the first survival study focusing specifically on older people with dementia admitted to NHs in France. The results provide valuable data on the length of stay in NHs and offer a detailed description of characteristics at admission.

The mean age of admission to NHs for the participants was 86 years, which is similar to that observed in France for the general population admitted (with and without dementia) [15]. The gender distribution in this study was 20% men and 80% women. This proportion of women is slightly higher than the general distribution observed in French NHs, where approximately 75% of residents are women, regardless of whether they have dementia [23]. The higher percentage of women in our sample may reflect the increased prevalence and progression of dementia in older women, who are more likely to reach the age of needing institutional care. The prevalence of diabetes appeared to be consistent with the epidemiological data available in France for the elderly population: 13% of older adults aged over 75 are diabetic [24], and 21% had hypercholesterolaemia. In contrast, public health studies show that 55% of people aged 65–74 have hypercholesterolaemia [25]. This discrepancy could be due to the age difference between the populations studied, as cholesterol levels often decline in very old age, especially in people with advanced dementia, due to factors such as malnutrition, weight loss, and the body’s natural changes in lipid metabolism as health deteriorates. As in our study, the prevalence of heart failure in older adults in France can be estimated at 12%, although this prevalence increases with age, reaching over 20% in people aged 80 and over, according to a national survey conducted in 2005 [26]. One notable difference concerns hypertension. This study reports a prevalence of 49% for hypertension, whereas the 3C cohort results report a higher prevalence of 62% in adults over 65 years of age [27].

The median survival duration for all subjects between admission to an institution and death was 39 months. Studies on the length of stays in NHs in France focus on all residents across the territory without distinguishing between people with and without dementia, and show an average duration (29 months) or median duration (31 months) that is shorter than the duration observed in this study [13,23]. As suggested in the literature, people with dementia admitted to NHs may have a longer length of stay than those admitted without dementia [17,28]. Being male, older, having more severe cognitive impairment on admission, and having more comorbidities were associated with decreased survival. AD is the most common form of dementia and a leading cause of death worldwide [29]. However, studies suggest that non-Alzheimer’s dementia is associated with higher mortality and shorter life expectancy than Alzheimer’s dementia [30]. This was not the case in this study. A low level of education is associated with an increased incidence of AD, but no study has been identified in the literature that suggests an association with mortality in people with AD or dementia [31], as confirmed in this study. No other factors with a significant effect on survival were identified among the observable variables. The results do not suggest that the risk of death might increase in parallel with an increase in the Katz score.

This study implies limitations. Participants admitted to NHs were from the Bordeaux centre of the 3C cohort, from which an ad hoc group was formed. This study involves a relatively small number of participants, which can be explained by the following aspects: (i) the fact that only participants from Bordeaux were included, as it is the only centre where the date of entry into an institution was documented; (ii) the average age of the study participants (73 years at the baseline), significantly lower than the typical age of admission to NHs in France (approximately 85 years) [23]. Although the sampling procedure aimed to obtain a representative population, the cohorts ultimately consist of volunteers who may differ from the general population. Compared with the general population aged 65 years and over in Bordeaux, 3C participants differed slightly in age, sex, and socioeconomic level distribution [18]. The presence of cancer may indeed have an impact on residents’ survival, but due to the limited number of cases in the sample, this variable could not be effectively included in the model. It is important to account for all symptomatic aspects of the disease and its overall severity, as these variables may influence survival. Only two clinical aspects reflecting the severity of dementia are considered (cognitive and functional capacity). Psycho-behavioural symptoms were not available and therefore could not be included in this study, which does not provide a comprehensive view of the severity of the disease and its progression. Inclusion of all aspects and types of dementia in our analyses would have provided additional insight to identify factors associated with mortality. This limits the interpretation of the results and the conclusion of a potential risk effect of the identified factors for people with dementia.

## 5. Conclusions

One of the major challenges in research on AD and other dementias is determining how best to support individuals with these debilitating conditions, especially after they have been admitted to long-term care facilities. These results suggest a longer duration of institutional care for people with dementia compared to the general French nursing home population, in a context where the number of people with dementia is expected to rise significantly in the coming years. The provision and organisation of care will become increasingly important challenges for public authorities. Preserving the dignity and quality of life of individuals with dementia, as well as that of their carers, are key drivers in the development of new public policies and interventions.

For future research, it will be crucial to include all aspects of dementia in assessments, including psycho-behavioural symptoms, to gain a more accurate understanding of the disease’s severity and its progression over time following admission to a long-term care facility. Quality of life is another essential aspect of dementia assessment, offering a more comprehensive picture of the disease’s impact on older adults and guiding interventions aimed at improving their overall well-being.

## Figures and Tables

**Table 1:** Description of the selected older adults with dementia at the admission in a nursing home (*n* = 201).

Characteristic	*n* = 201
Age *^1^	86 (5)
Gender	
Woman	160 (80%)
Man	41 (20%)
Marital Status (unknown *n* = 30)	
Divorced or single	25 (15%)
Married or in a relationship	38 (22%)
Widowed	108 (63%)
Level of Education (unknown *n* = 2)	
No diploma, primary and short secondary education	130 (65%)
Long secondary education	25 (13%)
Short and long higher education levels	44 (22%)
Dependency (Lawton) (unknown *n* = 55)	
Dependent	140 (96%)
Independent	6 (4%)
Dependency (Katz scale score) *^1^ (unknown *n* = 56)	3 (3)
Severe cognitive impairment (MMSE) *^1^ (unknown *n* = 63)	17 (7)
Type of Dementia (unknown *n* = 4)	
Alzheimer’s disease	139 (71%)
Others	58 (29%)
Number of drugs*^1^ (unknown *n* = 40)	7 (3)
Number of Comorbidities ^*2^ (unknown *n* = 28)	
None	54 (31%)
1	65 (38%)
2	44 (25%)
3 or more	10 (6%)
Diabetes (unknown *n* = 4)	26 (13%)
Cancer *^3^ (unknown *n* = 9)	11 (6%)
Hypertension (unknown *n* = 5)	93 (47%)
Cardiac rhythm disorders (unknown *n* = 8)	38 (20%)
Peripheral artery disease (unknown *n* = 7)	7 (4%)
Heart failure (unknown *n* = 7)	20 (10%)
Hypercholesterolaemia (unknown *n* = 9)	39 (20%)

*^1^ Mean (SD). *^2^ Among the seven following comorbidities: hypertension, cardiac rhythm disorders, peripheral artery disease, heart failure, diabetes, cancer, and hypercholesterolaemia. *^3^ Monitored or treated for cancer within the last three years.

## Data Availability

Restrictions apply to the availability of these data. They were obtained from the 3C study team and are available with the permission of the 3C team and their ethics as well as scientific committees.

## Supplementary Materials

The following supporting information can be downloaded at: https://www.mdpi.com/article/10.3390/geriatrics9060149/s1, Figure S1: Survival curve for the subsample of older adults with dementia (a), and stratified by gender (b), *n* = 119; Table S1: Description of the sample at the baseline for the Kaplan–Meier stratified on MMSE and Katz (*n* = 119); Table S2: Factors associated with mortality among older adults with dementia admitted in a nursing home, results of the Cox model (*n* = 108).
