# Supplementary material for: Mortality in Newly Admitted Nursing Home Older Adults with Dementia in France: A Post Hoc Analysis from an Observational Study in the Bordeaux Region
Source: Geriatrics (Basel). 2024 Nov 13;9(6):149. doi: 10.3390/geriatrics9060149 (PMC11586958; doi:10.3390/geriatrics9060149)
Supplement: Supplementary file 1 [file geriatrics-09-00149-s001.zip › geriatrics-3192052-supplementary.pdf]

Table S1. Baseline description of the sample for Kaplan-Meier analysis stratified by MMSE and Katz scores (n = 135)

**Characteristics**	**N = 135 *[1]**
**Age**	86(4)
**Gender**	
Woman	79% (107)
Man	21% (28)
**Marital status**	
Divorced or single	18% (23)
Married or in a relationship	25% (33)
Widowed	57% (75)
Unknown	4
**Level of education**	
No diploma, primary and short secondary education	68% (91)
Long secondary education	10% (13)
Short and long higher education levels	22% (30)
Unknown	1
**Dependency (Lawton)**	95% (127)
Dependent	5% (6)
Independent	
Unknow	2
**Dependency (Katz)**	3 (3)
**Cognitive impairment (MMSE)**	17 (7)
**Type of dementia**	
Alzheimer's disease	67% (89)
Others	33% (44)
Unknown	2
**Number of drugs (unknown = 16)**	7 (3)
**Number of comorbidities *[2]**	
None	31% (37)
1	34% (41)
2	29% (34)
3 or more	6% (7)
Unknown	16
**Diabetes**	15% (20)
Unknown	1
**Cancer *[3]**	7% (9)
**Hypertension**	48% (64)
Unknown	2
**Cardiac rhythm disorders**	24% (32)
Unknown	4
**Peripheral artery disease**	2% (3)
Unknown	4
**Heart failure**	12% (16)
Unknown	2
**Hypercholesterolemia**	22% (29)
Unknown	4

*[1] Mean (SD); %(n); *[2] Among the seven following comorbidities: hypertension, cardiovascular disease (cardiac rhythm disorders, peripheral artery disease, heart failure), diabetes, cancer, hypercholesterolemia; *[3] Monitored or treated for cancer within the last three years.

Table S2. Factors associated with mortality among demented nursing home residents, results of the Cox model (*n* = 118).

Variable	HR	95% CI	*p*-value
Gender – Man vs woman	3.13	1.88–5.20	1.07e-05 ***
Age	1.08	1.02–1.14	0.0047 **
MMSE – severe vs. mild to moderate	1.61	0.91–2.84	0.1036
Hypertension	1.52	1.00–2.31	0.0514 **.**
Number of comorbidities (≥3 vs ≤2)	4.10	1.82–9.24	0.0006 ***

CI - lower and upper bound 95% confidence intervals; *** 0.001; ** 0.01; * 0.05; **.** 0.1

(A)

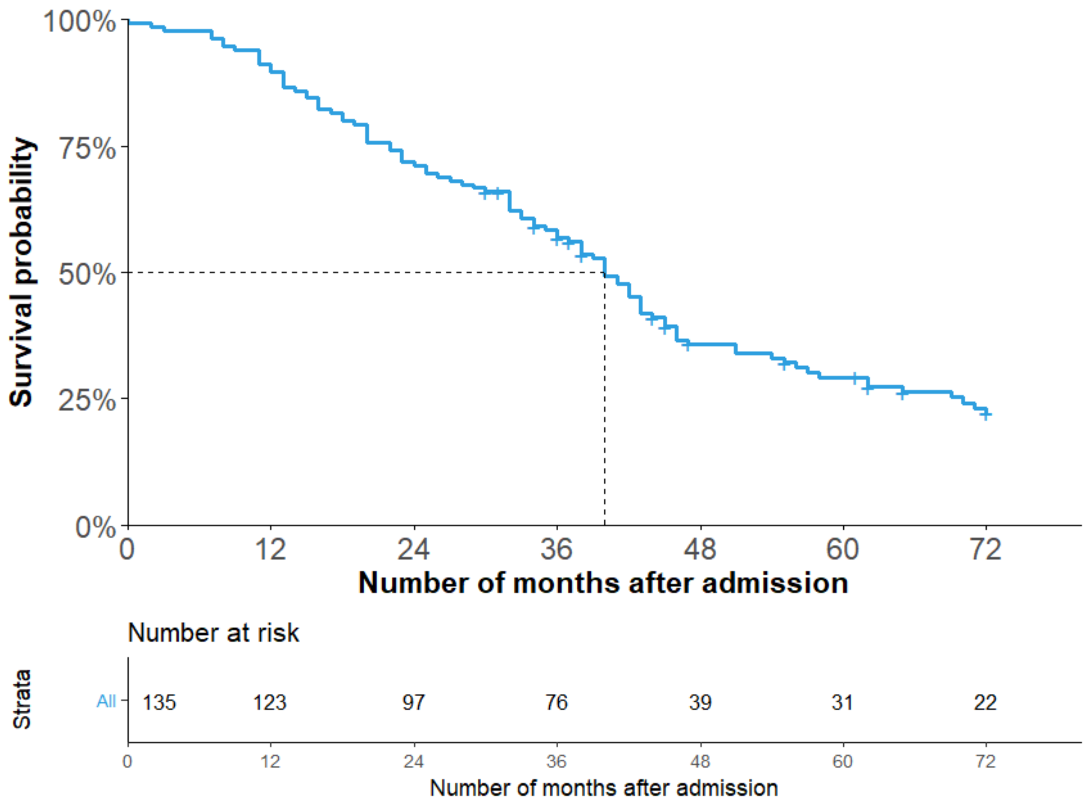

(B)

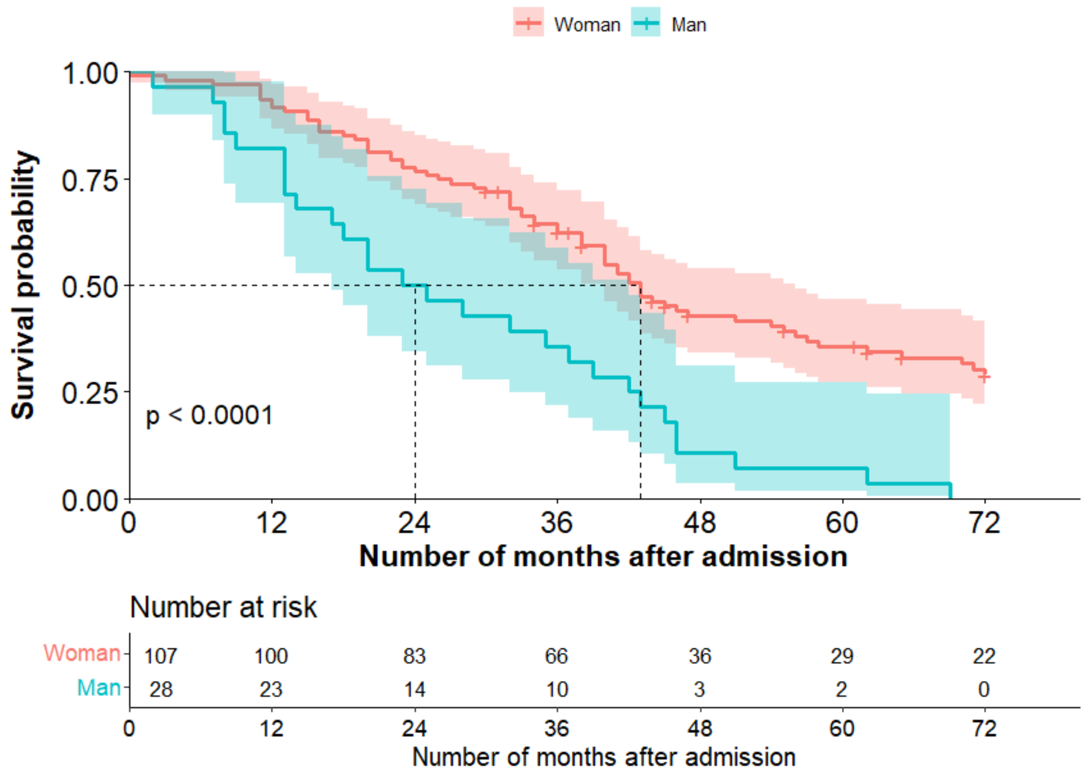

Figure S1. Survival curve for the subsample of older adults with dementia (**A**), and stratified by gender (**B**), $n = 135$.
